# Dual targeting of acute myeloid leukemia progenitors by catalytic mTOR inhibition and blockade of the p110α subunit of PI3 kinase

**DOI:** 10.18632/oncotarget.3509

**Published:** 2015-03-10

**Authors:** Marco Colamonici, Gavin Blyth, Diana Saleiro, Amy Szilard, Meghan Bliss-Moreau, Francis J. Giles, Jessica K. Altman, Elspeth M. Beauchamp, Leonidas C. Platanias

**Affiliations:** ^1^ Robert H. Lurie Comprehensive Cancer Center of Northwestern University, Chicago, IL, USA; ^2^ Division of Hematology-Oncology, Department of Medicine, Feinberg School of Medicine, Northwestern University, Chicago, IL, USA; ^3^ Division of Hematology-Oncology, Department of Medicine, Jesse Brown VA Medical Center, Chicago, IL, USA

**Keywords:** AML, mTOR signaling, PI3 kinase

## Abstract

The mammalian target of rapamycin (mTOR) and phosphoinositide-3-kinase (PI3K) pathways are often aberrantly activated in acute myeloid leukemia (AML) and play critical roles in proliferation and survival of leukemia cells. We provide evidence that simultaneous targeting of mTOR complexes with the catalytic mTOR inhibitor OSI-027 and of the p110α subunit of PI3K with the specific inhibitor BYL-719 results in efficient suppression of effector pathways and enhanced induction of apoptosis of leukemia cells. Importantly, such a combined targeting approach results in enhanced suppression of primitive leukemic progenitors from patients with AML. Taken together, these findings raise the possibility of combination treatments of mTOR and p110α inhibitors as a unique approach to enhance responses in refractory AML.

## INTRODUCTION

Acute myeloid leukemia (AML) is a highly fatal disease, as many patients do not respond to therapy or eventually relapse [1-3]. For older patients or patients with co-morbities who cannot tolerate intensive chemotherapy, the treatment options are particularly limited [4]. There is undoubtedly a need for new therapeutic regimens and innovative approaches to overcome resistance in AML. The disease has been associated with dysregulation and constitutive activation of the mammalian target of rapamycin (mTOR) and phosphoinositide-3-kinase (PI3K) signaling pathways, which promote aberrant cell growth and survival, and induce anti-apoptotic responses [5-8].

mTOR exists in two distinct complexes, mTOR complex 1 (mTORC1) and mTOR complex 2 (mTORC2). Each complex has distinct characteristics and downstream effectors, resulting in distinct functional outcomes. Activation of mTORC1 (a complex composed of mTOR, Raptor, PRAS40, mLST8 and DEPTOR) plays a central role in regulating initiation of mRNA translation and autophagy through its downstream targets 4E-BP1, S6K and ULK1 [9-11]. Activation of mTORC2 (a complex formed by mTOR, RICTOR, SIN1, mLST8, PROTOR and DEPTOR) regulates the pro-survival family of AGC kinases, including the kinase AKT, leading to effects on cell metabolism, survival, proliferation, and cytoskeletal rearrangement [9-11]. Specific mTORC1 inhibition has been extensively studied using rapamycin, however, recent evidence suggests the existence of rapamycin-insensitive mTORC1 complexes [12-15]. More recently, a new generation of catalytic mTOR inhibitors has been developed to target both mTOR complexes [12, 13, 16]. These mTOR inhibitors act by binding to the ATP-binding site of mTOR and thus block the activities of both mTORC1 and mTORC2 [12, 13, 16]. We have previously reported that dual targeting of mTORC1 and mTORC2 with OSI-027 results in enhanced antileukemic responses as compared to treatment with the classic mTORC1 inhibitor, rapamycin [17].

PI3K has also been identified as an important target for the treatment of several types of cancer, including AML [18-23]. Abnormal activation of the PI3K signaling pathway has been directly correlated to oncogenic activity, due to its prominent role in mediating cellular growth and survival [18, 20, 22]. There are three known classes of PI3K [22, 24]. Class-IA PI3K are heterodimers composed by a regulatory subunit, p85, and a catalytic subunit p110 [22, 24, 25]. Mutations leading to constitutive activation of the p110α subunit have been found in several types of cancer [18-22, 24]. PI3K is activated in AML, however, the mechanism is unknown, as mutations in PI3K isoforms have not been found [23, 26]. BYL-719 is a specific class-IA PI3K inhibitor, which acts by binding the ATP binding domain of the p110α subunit [27]. Recently, BYL-719 has been reported to have significant activity against tumors carrying mutations in the p110α subunit of PI3K [28, 29]. However, BYL-719 has achieved only modest effects as a single agent in malignancies with non-mutated PI3K [21, 29-31].

In the present study, we sought to evaluate the effects of combined targeting of AML cells, using a dual mTOR inhibitor, OSI-027, and a p110α subunit inhibitor, BYL-719. Our studies provide evidence that the OSI-027/BYL-719 combination induces potent and synergistic anti-leukemic responses in several AML cell lines with diverse molecular characteristics and in primary leukemic progenitors (CFU-L) from AML patients.

## RESULTS

**Figure 1:**
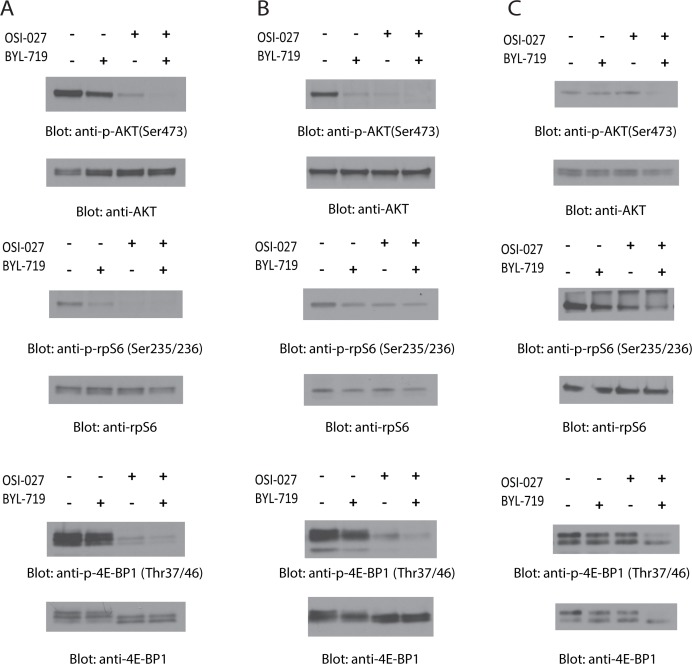
Dual targeting of PI3K and mTOR by BYL-719 and OSI-027 results in enhanced inhibition of activation of PI3K and mTOR pathways (A) U937, (B) MM6 and (C) Kasumi-1 cells were treated with BYL-719 and OSI-027, alone or in combination for 90 minutes. Equal amounts of total cell lysates were subjected to SDS-PAGE and immunoblotted with antibodies against the phosphorylated forms of AKT (pSer-473), rpS6 (pSer-235/236), and 4E-BP1 (pThr-37/46), as indicated. The corresponding blots were stripped and reprobed with antibodies against total AKT, rpS6, and 4E-BP1, as indicated.

In initial studies, we examined the effects of BYL-719 and OSI-027, alone and/or in combination, on the phosphorylation of PI3K and mTOR downstream targets. Using different AML cell lines (U937, MM6 and Kasumi-1), we analyzed the effects of these agents on the phosphorylation of AKT on serine 473 (Ser473), a marker of mTORC2 activity, and the phosphorylation of S6 ribosomal protein (rpS6) and eukaryotic translation initiation factor 4E-binding protein 1 (4E-BP1), markers of mTORC1 activity. Treatment of cells with BYL-719 or OSI-027 alone resulted in a decrease in the phosphorylation levels of AKT, rpS6, and 4E-BP1 in most cases (Fig. 1A, 1B and 1C). Addition of BYL-719 did not result in significant differences from the OSI-027-dependent inhibition of phosphorylation of rpS6 in U937 or MM6 cells (Fig. 1A and 1B). However, in most cases, combined treatment with BYL-719 and OSI-027 resulted in more potent reduction of the phosphorylation levels of the different proteins (Fig. 1A, 1B and 1C), indicating a synergistic effect on these effectors of the PI3K/mTOR pathways. Notably, in Kasumi-1 cells neither BYL-719 nor OSI-027 alone suppressed phosphorylation of AKT on serine 473, but the combination of the two agents resulted in potent suppressive effects (Fig. 1C).

**Figure 2:**
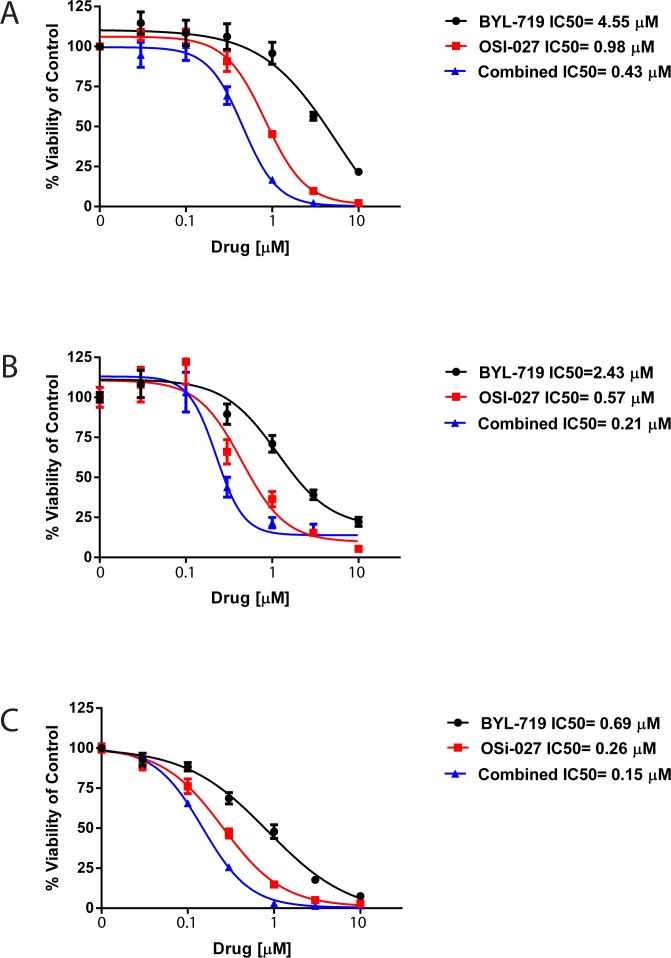
BYL-719 and OSI-027 synergize to inhibit the viability of AML cells (A) U937, (B) MM6, (C) Kasumi-1 cells were plated in 96 well plates and treated with BYL-719 and OSI-027 alone and in combination, as indicated, for 72 hours. Viability was assessed using a WST-1 assay. Data are expressed as percentage of vehicle-treated cells (control). Shown are means and standard errors of five (A) or four (B and C) independent experiments.

In subsequent experiments we evaluated the effects of combination treatment with BYL-719 and OSI-027 versus single-drug treatments on viability/cell proliferation of AML cell lines. As shown in Fig. 2, single-drug treatments resulted in inhibition of cell proliferation. However, combination treatment with BYL-719 and OSI-027 strongly enhanced the growth inhibitory effects of each drug alone in all AML cell lines. The half maximal inhibitory concentration (IC_50_) and the combination index (CI) values were calculated for the combination and single-drug treatments. CI values lower than 1.0 indicate synergistic effects against proliferation by the combination treatment, values greater than 1.0 indicate antagonistic effects, and values equal to 1.0 indicate additive effects. The IC_50_ of BYL-719 and OSI-027 on U937 cells were 4.55μM and 0.98μM, respectively, whereas the IC_50_ of the combination treatment was 0.43μM (Fig. 2A). The CI for the combination treatment was 0.54, indicating a synergistic effect against proliferation of U937 cells. Similar results were observed for the MM6 (Fig. 2B) and Kasumi-1 (Fig. 2C) cell lines subjected to single-drug and combination treatments. The IC_50_ for BYL-719 and OSI-027 on the MM6 cell line were 2.43μM and 0.57μM, respectively, while the combination treatment resulted in an IC_50_ of 0.21μM and a CI value of 0.45 again showing synergism (Fig. 2B). Treatment of Kasumi-1 cells revealed an IC_50_ of 0.69μM for BYL-719, 0.26μM for OSI-027, and 0.15μM for the combination treatment (Fig. 2C). The CI value on this cell line was 0.78, which also indicates synergism between BYL-719 and OSI-027.

**Figure 3:**
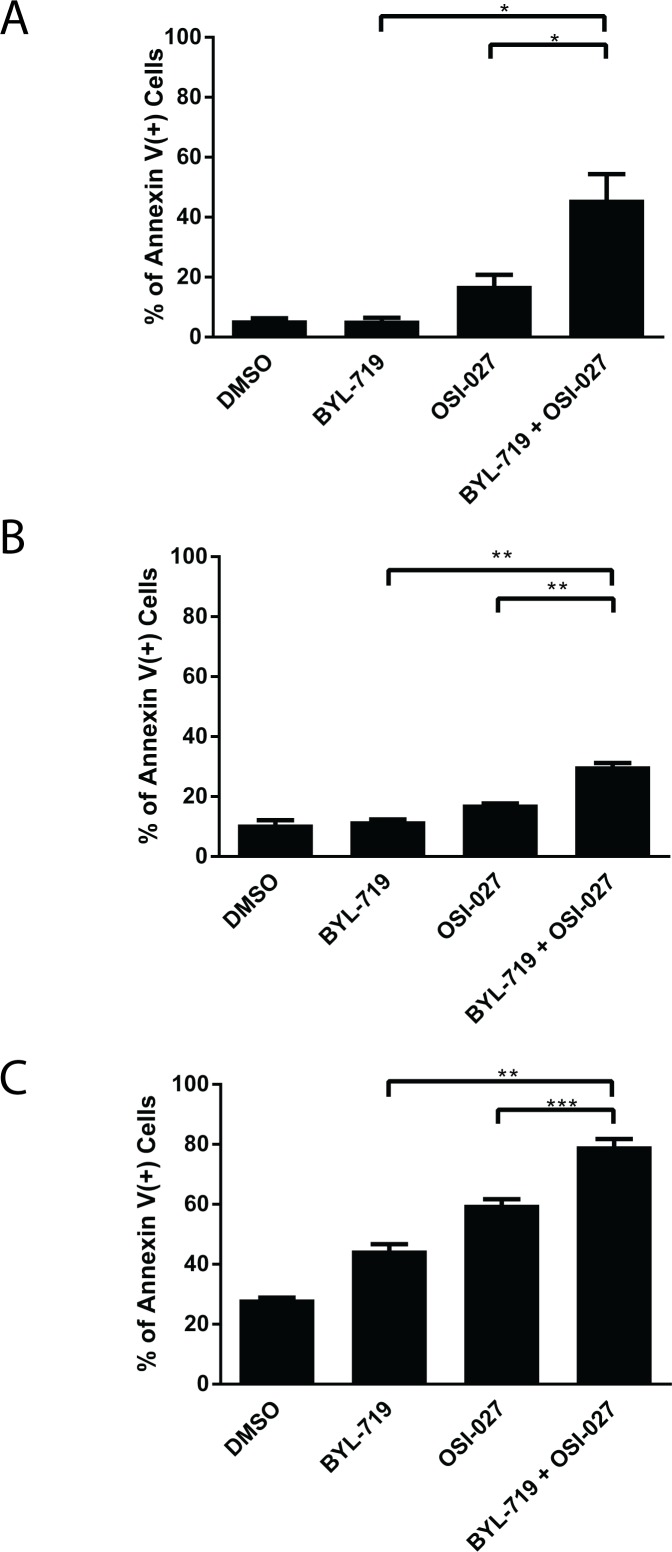
The combination of BYL-719 and OSI-027 results in enhanced induction of apoptosis in AML cell lines (A) U937, (B) MM6 and (C) Kasumi-1 cells were treated with BYL-719 or OSI-027 alone or in combination for 72 hours, as indicated. The percentage of apoptosis was determined by flow cytometry using DAPI/Annexin V staining. Shown are means and standard errors of three independent experiments. * p < 0.05, ** p < 0.01, *** p < 0.001 using a paired two-tailed t-test.

To determine whether the potent and synergistic effects of the combined treatment with BYL-719 and OSI-027 correlated with induction of pro-apoptotic responses, we next measured the induction of cellular apoptosis using flow cytometry analysis for double staining with annexin V/DAPI. The combination treatment of BYL-719 and OSI-027 resulted in a statistically significant increase in the percentage of apoptotic cells, when compared to either inhibitor alone, in all AML cell lines (Fig. 3).

**Figure 4:**
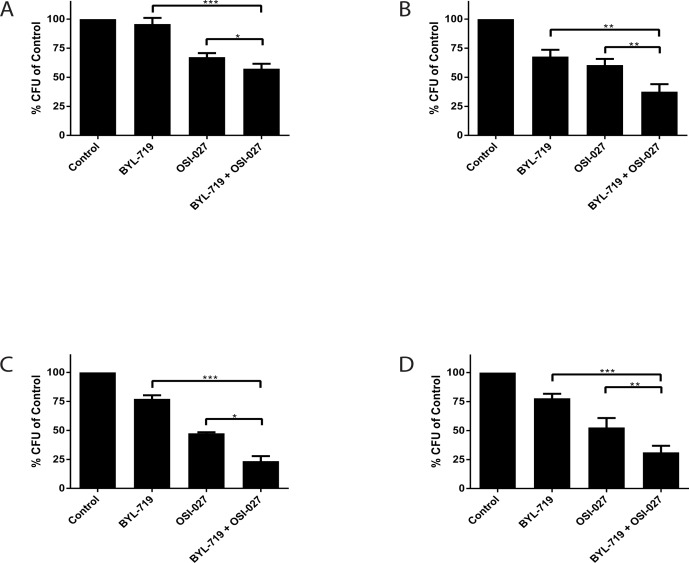
Enhanced antileukemic effects of the combination of BYL-719 and OSI-027 (A) U937 (B) MM6 and (C) Kasumi-1 cells were plated in clonogenic assays in methylcellulose in the presence of BYL-719 and OSI-027 alone or in combination, as indicated. Shown are means and standard errors of five (A and B) or three independent experiments. (D) Primary leukemic progenitor colony formation (CFU-L) from different AML patients was assessed in clonogenic assays in methylcellulose, in the presence of BYL-719, OSI-027 or both agents, as indicated. Shown are the means and standard errors of 5 experiments performed with samples from 5 different AML patients. * p < 0.05, ** p < 0.01, *** p <0.001 using a paired two-tailed t-test.

To evaluate the anti-leukemic effects induced by the combination treatment with BYL-719 and OSI-027 in a more pathophysiologically relevant system, we assessed effects on leukemic progenitors (CFU-L) using clonogenic assays in methylcellulose. The combined treatment of BYL-719 and OSI-027 resulted in greater inhibition of CFU-L colony growth of leukemic precursors in all lines (Figs. 4A, 4B, and 4C). Importantly, the combination resulted in enhanced suppression of primary leukemic precursors from patients with AML (Fig. 4D), suggesting that the combination of these agents may provide a better strategy to target primitive leukemic precursors.

## DISCUSSION

Constitutive activation of PI3K and mTOR pathways plays a key role in uncontrolled growth and survival of malignant cells [9-11, 18, 20, 22]. Because of such observations, these pathways have been identified as important targets for the treatment of several malignancies, including AML [9, 18-23]. We have previously shown that dual targeting of mTORC1 and mTORC2 with OSI-027 results in more potent antileukemic effects than treatment with rapamycin alone [17]. We have also established that catalytic mTOR inhibition enhances the suppressive effects of cytarabine [17], while other studies have shown that catalytic mTOR inhibitors are potentially less immunosuppressive than rapamycin [32]. Taken together, the results of these studies have suggested that catalytic mTOR inhibitors may be more optimal agents for the treatment of malignancies as compared to rapalogs, exhibiting more potent antineoplastic effects and less immunosuppression.

Pharmacological inhibition of PI3K using the LY294002 inhibitor was shown to block proliferation of primary AML blasts through inhibition of AKT-induced survival pathways [33]. LY2940002, however, has limited clinical potential due to toxicity problems and off-target effects [34]. Inhibition of PI3K by BYL-719 showed limited efficacy as a single agent in a RAS-driven model of AML [21]. Here, we present data that BYL-719 has modest antileukemic effects as a single agent on AML cells. The relative modest effects of PI3K inhibition alone can be in part explained by the fact that although PI3K activity is inhibited, activation of its downstream effectors, including AKT and mTORC1, is not completely blocked [28]. This could be explained via engagement of mTOR by signaling loops that circumvent the inhibition of PI3K by BYL-719 [28]. Notably, in pancreatic neuroendocrine tumors targeting of both PI3K and mTOR was required to generate inhibitory effects on cells that were resistant to the mTORC1 inhibitor RAD001 [31]. Regarding limitations of single mTOR or PI3K targeting in AML cells, it has been previously demonstrated that mTOR inhibition leads to activation of PI3K signaling via upregulation of IGF-1 [35]. Additionally, combinations of specific mTORC1 inhibitors with PI3K inhibitors may not be effective as a potential feedback loop involving mTORC2 activation by growth factors may contribute to survival of cancer cells [9-11]. Inhibition of mTORC1 or PI3K alone also effects cytokine production by AML cells that may induce negative feedback loops [36]. In addition, mTORC1 inhibition can result in activation of MAPK signaling, independently of PI3K [37]. Thus, combined targeting using PI3K and catalytic mTOR inhibitors provides a better strategy to prevent activation of feedback loops that lead to resistance to treatment. It should be noted that AML cells always express the PI3K isoform p110δ and heterogeneously express p110α and p110β [23]. Previously, p110δ selective inhibitors exhibited significant anti-leukemic effects on primary AML patient samples [38, 39]. Hence, future studies should assess the differences between targeting p110α and p110δ isoforms in terms of anti-oncogenic activity in AML. These studies should also address if inhibition of one isoform results in leukemic cell survival mechanisms involving compensation by activation of the other isoforms.

In the present study, we provide evidence that combination of BYL-719 and OSI-027 results in more potent inhibition of phosphorylation of mTOR downstream targets and in synergistic inhibition of leukemic cell viability and induction of apoptosis. In addition, we establish that combination of BYL-719 with OSI-027 is much more effective than treatment with each inhibitor alone in blocking the survival of primitive leukemic progenitors from AML patients *in vitro*. Studies from other groups have shown that dual PI3K-mTORC1/2 inhibitors exhibit antileukemic properties in AML [40-42], and are consistent with our findings. Altogether, the results of our studies support the combination of PI3K and mTOR inhibitors as a potential novel approach for the treatment of AML that warrants clinical study.

## MATERIALS AND METHODS

### Cells and reagents

The U937 and Kasumi-1 human AML cell lines were purchased from American Type Cell Culture (ATCC). MM6 cells were purchased from DSMZ. U937 cells were cultured in RPMI 1640 medium supplemented with 10% fetal bovine serum (FBS), while the Kasumi-1 cell line was cultured in ATCC modified RPMI 1640 medium supplemented with 20% FBS. MM6 cells were cultured in RPMI 1640 medium supplemented with 10 μg/ml human insulin, 1 mM sodium pyruvate, 1 mM nonessential amino acids, and 10% FBS. BYL-719 and OSI-027 were both purchased from ChemieTek.

### Cell lysis and immunoblotting

Cells were lysed in phosphorylation lysis buffer (50 mM HEPES, pH 7.9; 150 mM NaCl; 4 mM Na Pyrophosphate; 1 mM EDTA, pH 8.0; 10 mM NaF; 0.5% Triton-X 100; 10% glycerol) with 1 mM PMSF, protease inhibitor cocktail set V (EMD Millipore) and PhosStop phosphatase inhibitor (Roche). Cell lysates were subject to SDS-PAGE and then transferred to Immobilon-P membranes (Millipore). Membranes were then subjected to blocking in 5% nonfat dry milk and 1% BSA in 1X TBST (20 mM Tris-HCL, pH 7.5; 150 mM NaCl; and 0.5% Tween 20) for 1 hour. All antibodies were purchased from Cell Signaling Technologies except the antibody against GAPDH (Millipore). Primary antibodies were added to the membrane in 1X TBST for 1 hour or overnight. The membrane was then washed 3 times in 1X TBST and HRP-linked anti-rabbit (GE Healthcare) or anti-mouse secondary antibody (Bio-Rad) was added for 1 hour. Blots were washed 3 times in 1X TBST and then developed using Amersham ECL western blotting detection reagent (GE Healthcare Life Sciences) or Immobilon western chemiluminescent HRP Substrate (Millipore) per the manufacturer's instructions. Chemiluminescence was detected using autoradiography film. Films were digitally scanned with Adobe Photoshop CS5 using a Canon CanoScan 8800F scanner. To assess the effects of combination treatment on the phosphorylation/activation of different mTOR effectors, U937 and MM6 cells were treated with BYL-719 or OSI-027 alone or in combination for 90 minutes, at final concentrations of 1μM and 0.5μM, respectively. Kasumi-1 cells were treated with BYL-719 or OSI-027 alone or in combination for 90 minutes, at final concentrations of 0.25μM and 0.15μM, respectively.

### Clonogenic leukemic progenitor assays in methylcellulose

These assays were performed as described in previous studies [17, 43]. Briefly, peripheral blood or bone marrow samples were obtained from patients with AML after obtaining informed consent approved by the Institutional Review Board of Northwestern University. Mononuclear cells were isolated by Ficoll-Hypaque (Sigma Aldrich) sedimentation. To assess the effects of drugs on leukemic progenitor (CFU-L) colony formation, cells were plated in methylcellulose (MethoCult™ H4534 Classic without EPO, Stem Cell Technologies), in the presence of BYL-719 or OSI-027 alone or in combination, at a final concentration of 1μM. For cell line-derived CFU-L colony formation, U937 and Kasumi 1 cells were incubated in methylcellulose with BYL-719 or OSI-027 alone or in combination, at final concentrations of 1μM and 5μM, respectively. MM6 cells were incubated in methylcellulose with BYL-719 or OSI-027 alone or in combination, at a final concentration of 1μM.

### Cell viability assays

Cellular viability was assessed by plating AML cell lines in triplicates in 96-well plates. Cells were treated with vehicle alone (DMSO), BYL-719, OSI-027, or combination of BYL-719 and OSI-027 at the indicated concentrations. 72 hours later, cell viability and proliferation were quantified using the WST-1 Reagent (Roche) according to the manufacturer's protocol. IC_50_ and CI values were calculated using Compusyn to determine whether drug interactions were additive, synergistic or antagonistic [44].

### Cell death assays

Cells were treated with vehicle (DMSO), BYL-719, OSI-027 or the combination of BYL-719 and OSI-027 for 72 hours. U937 and MM6 cells were treated with BYL-719 or OSI-027 alone or in combination, at a final concentration of 5μM. Kasumi-1 cells were treated with these agents at a final concentration of 1μM. Cells were stained with Annexin V and 4′-6-diamidino-2-phenylindole (DAPI)-DNA staining according to the manufacturer's instructions (BD Biosciences). Flow cytometric analyses were performed on stained cells using a LSRFortessa flow cytometer (BD Biosciences). Cell gating and analyses were done using FlowJo software (Tree Star, Ashland, OR).

### Statistics

All statistics were performed using Prism Graphpad 6.0 for PC.
